# Supplementary material for: Genetic Nurture Effects on Type 2 Diabetes Among Chinese Han Adults: A Family-Based Design
Source: Biomedicines. 2025 Jan 7;13(1):120. doi: 10.3390/biomedicines13010120 (PMC11761613; doi:10.3390/biomedicines13010120)
Supplement: Supplementary file 1 [file biomedicines-13-00120-s001.zip › biomedicines-3397396 - Supplementary Material File 1.pdf]

Supplementary material

1.1 Study design and collection of pedigrees

The Fangshan/Family-based Ischemic Stroke Study in China (FISSIC) project is a community-based, hospital-centered genetic epidemiological study of multiple chronic diseases. FISSIC initially planned to collect only ischemic stroke pedigrees, as stated in pioneering protocol paper [1]. Then it subsequently extended it scope to include pedigrees for Type 2 diabetes and hypertension. The protocol and corresponding researches systematically described these efforts [2-4]. The FISSIC program was conducted in Fangshan District, a rural area located southwest of Beijing, China (39°30′~39°55′ N, 115°25′~116°15′ E) since 2005. The first phase is scheduled to be finished in August 2017. The study design was an ischemic stroke, type 2 diabetes, hypertension spectrum-based family study involving the proband, their siblings, and their parents. As for pedigrees collection, proband-initiated contact method was used. Specifically, probands were defined as the individual with previously diagnosed ischemic stroke, type 2 diabetes, hypertension in community hospital and verified at the central hospital. Their parents' and siblings' information were obtained from probands under informed consent. Obtaining the informed consent, parents and siblings were recruited and surveyed.

1.2 Genotyping

Samples are sent to a central laboratory for processing, testing and genotyping. As a result, 743,722 SNPs were genotyped in each sample. QC was performed on each sample. The individuals were retained if they achieved the following criteria: > 95% variant call rate, consistency between genotyped sex and the investigated sex, heterozygosity < 3 standard deviations from the average, consistency between inferenced kinship using identity-by-descents (IBDs) and investigated kinship, < 5% Mendelian errors and no significant deviation from principal components of the ancestral background. We also performed QC on each call set, SNP were retained if they achieved the following criteria: > 95% sample call rate, Hardy–Weinberg equilibrium $P > 1 \times 10^{-6}$, minimum allele frequency (MAF) > 1%, and < 10% Mendelian errors. All the QCs were conducted via PLINK 1.9 software (https://www.cog-genomics.org/plink/) [5]. A total of 500,599 SNPs passed the QC and were used for genotype imputation.

1.3 Parental genotype imputation

We used snipar to impute the missing parent genotype using full sibling and parent–offspring pairs genotype [6].

Given the genotypes of a sibling pair and the IBDs state of alleles (which alleles are shared by descent from the parents), snipar estimates the sum of the parents' genotypes of sibling, and imputed the unobserved parental genotype using the population frequency of allele 1. When in IBD=1 (the siblings share one allele IBD), the alleles not shared are known unless both siblings are heterozygous. When both are heterozygous, information from neighboring phased SNPs can be used to resolve the uncertainty. However, without phased data, imputation can proceed by averaging over the two possibilities (shared allele is 0 versus shared allele is 1).

As for parent–offspring pairs, genotype of a proband's parent can be imputed given the observations of the proband and another's parent genotypes based on Mendelian laws, unless both proband and known parental are heterozygous. If proband and known parental are heterozygous (for example, they are all Aa), information from neighboring phased SNPs is needed to resolve the uncertainty. Without phased data, the unobserved paternal genotype can be imputed by averaging over the two possible inheritance patterns (AA or Aa).

1.4 Statistical analysis

Definition of genomic loci and SNP mapping

According to FUMA, SNPs achiveve whole genomic significant and independent of each other at LD $r^2 < 0.6$ were identified as significant independent SNPs [7]. Those SNPs independent of each other at LD $r^2 < 0.1$ were then selected as lead SNPs. The border for a genomic locus was defined as a region containing all candidate SNPs in LD ($r^2 > 0.6$) with at least a lead SNP. Candidate SNPs were merged into a genomic locus if the distances between them were less than 250 Kb.

FUMA assigns the lead SNPs and additional independent SNPs in each genomic risk locus to their mapped genes not only based on genomic position, but also on three kind of Function annotation method, namely, combined annotation dependent depletion (CADD) score, probability of regulatory functionality (Regulome DB score), and transcription/regulatory effects from chromatin states (Minimum chromatin state) [8-10]. CADD scores the deleteriousness of an SNP by summarizing 63 Function annotations with a support vector machine, SNP with $CADD\ score\ \geq 12.37$ is more likely to be pathogenic[8]. Regulome DB, ranging from 1 to 7, assesses the possibility of transcription factor binding and gene expression being affecting by a SNP based on ENCODE and other sources. SNPs with $Regulome\ DB\ score\ \leq 2$ were defined as functional signals [9]. Minimum chromatin state can predict the accessibility of chromatin region, and was established by a multivariate hidden Markov model with 15 categorical states on the basis of 5 histone modification marks for 127 epigenomes [10]. A score of 8-15 indicates the closed chromatin state. On the 1-7 scale, lower score indicates more open chromatin states, suggesting the higher DNA regulatory potential of the genomic region where SNP was located.

Pathway-based analysis using GSA-SNP2

GSA-SNP2 accepts human GWAS summary data (SNP, P values) and outputs pathway gene sets 'enriched' with genes associated with the given phenotype [11]. GSA-SNP2 employs the Z-statistic of the random set model for evaluating gene-sets [12]. Specifically, it calculated the gene scores adjusted for the SNP counts for each gene using a monotone cubic spline trend, and then obtain the gene sets p-value from the right-tailed test under the assumption that pathway statistic has standard normal distribution (Each pathway gene-set is assumed to be a random collection of genes from the genome). Significance of a gene-set is determined by q value which is based on the trend curve adjusted gene scores. Benjamini–Hochberg method is used for the multiple testing correction. This gene set information is from different databases and has been integrated into a dataset from the Human Molecular Signatures Database (MSigDB). We download the curated gene sets (MSigDB c2.all.v2024.1. Hs.symbols.gmt,

accessed on August 24, 2024) and ontology gene sets (MSigDB c5.all.v2024.1.Hs.symbols.gmt, accessed on August 24, 2024) from MSigDB database (https://www.gsea-msigdb.org/gsea/msigdb/human/collections.jsp) and used for annotation.

Reference

1. Tang, X.; Hu, Y.; Chen, D.; Zhan, S.; Zhang, Z.; Dou, H. The Fangshan/Family-based Ischemic Stroke Study In China (FISSIC) protocol. *BMC Med Genet* **2007**, *8*, 60, doi:10.1186/1471-2350-8-60.
2. Wu, N.; Tang, X.; Wu, Y.; Qin, X.; He, L.; Wang, J.; Li, N.; Li, J.; Zhang, Z.; Dou, H.; et al. Cohort profile: the Fangshan Cohort Study of cardiovascular epidemiology in Beijing, China. *J Epidemiol* **2014**, *24*, 84-93, doi:10.2188/jea.je20120230.
3. Wu, J.; Wang, X.; Chen, H.; Yang, R.; Yu, H.; Wu, Y.; Hu, Y. Type 2 Diabetes Risk and Lipid Metabolism Related to the Pleiotropic Effects of an ABCB1 Variant: A Chinese Family-Based Cohort Study. *Metabolites* **2022**, *12*, doi:10.3390/metabo12090875.
4. Yin, Q.; Sun, K.; Xiang, X.; Juan, J.; Cao, Y.; Song, J.; Yang, Y.; Shi, M.; Tian, Y.; Liu, K.; et al. Identification of Novel CXCL12 Genetic Polymorphisms Associated with Type 2 Diabetes Mellitus: A Chinese Sib-Pair Study. *Genet Test Mol Biomarkers* **2019**, *23*, 435-441, doi:10.1089/gtmb.2018.0149.
5. Purcell, S.; Neale, B.; Todd-Brown, K.; Thomas, L.; Ferreira, M.A.; Bender, D.; Maller, J.; Sklar, P.; de Bakker, P.I.; Daly, M.J.; et al. PLINK: a tool set for whole-genome association and population-based linkage analyses. *Am J Hum Genet* **2007**, *81*, 559-575, doi:10.1086/519795.
6. Young, A.I.; Nehzati, S.M.; Benonisdottir, S.; Okbay, A.; Jayashankar, H.; Lee, C.; Cesarini, D.; Benjamin, D.J.; Turley, P.; Kong, A. Mendelian imputation of parental genotypes improves estimates of direct genetic effects. *Nat Genet* **2022**, *54*, 897-905, doi:10.1038/s41588-022-01085-0.
7. Watanabe, K.; Taskesen, E.; van Bochoven, A.; Posthuma, D. Functional mapping and annotation of genetic associations with FUMA. *Nature Communications* **2017**, *8*, 1826, doi:10.1038/s41467-017-01261-5.
8. Kircher, M.; Witten, D.M.; Jain, P.; O'Roak, B.J.; Cooper, G.M.; Shendure, J. A general framework for estimating the relative pathogenicity of human genetic variants. *Nat Genet* **2014**, *46*, 310-315, doi:10.1038/ng.2892.
9. Boyle, A.P.; Hong, E.L.; Hariharan, M.; Cheng, Y.; Schaub, M.A.; Kasowski, M.; Karczewski, K.J.; Park, J.; Hitz, B.C.; Weng, S.; et al. Annotation of functional variation in personal genomes using RegulomeDB. *Genome Res* **2012**, *22*, 1790-1797, doi:10.1101/gr.137323.112.
10. Kundaje, A.; Meuleman, W.; Ernst, J.; Bilenky, M.; Yen, A.; Heravi-Moussavi, A.; Kheradpour, P.; Zhang, Z.; Wang, J.; Ziller, M.J.; et al. Integrative analysis of 111 reference human epigenomes. *Nature* **2015**, *518*, 317-330, doi:10.1038/nature14248.
11. Yoon, S.; Nguyen, H.C.T.; Yoo, Y.J.; Kim, J.; Baik, B.; Kim, S.; Kim, J.; Kim, S.; Nam, D. Efficient pathway enrichment and network analysis of GWAS summary data using GSA-SNP2. *Nucleic Acids Res* **2018**, *46*, e60, doi:10.1093/nar/gky175.
12. Newton, M.A.; Quintana, F.A.; den Boon, J.A.; Sengupta, S.; Ahlquist, P. Random-set

methods identify distinct aspects of the enrichment signal in gene-set analysis. *The Annals of Applied Statistics* **2007**, *1*, 85-106, 122.
